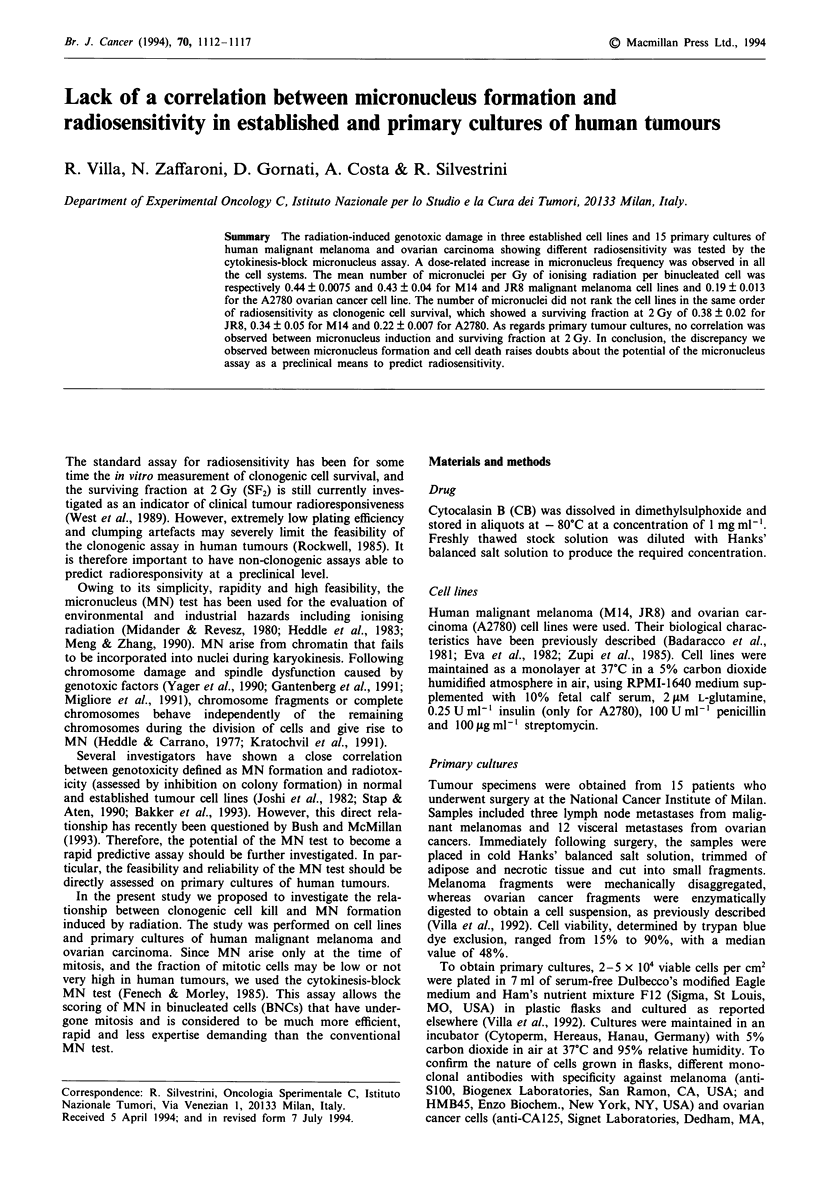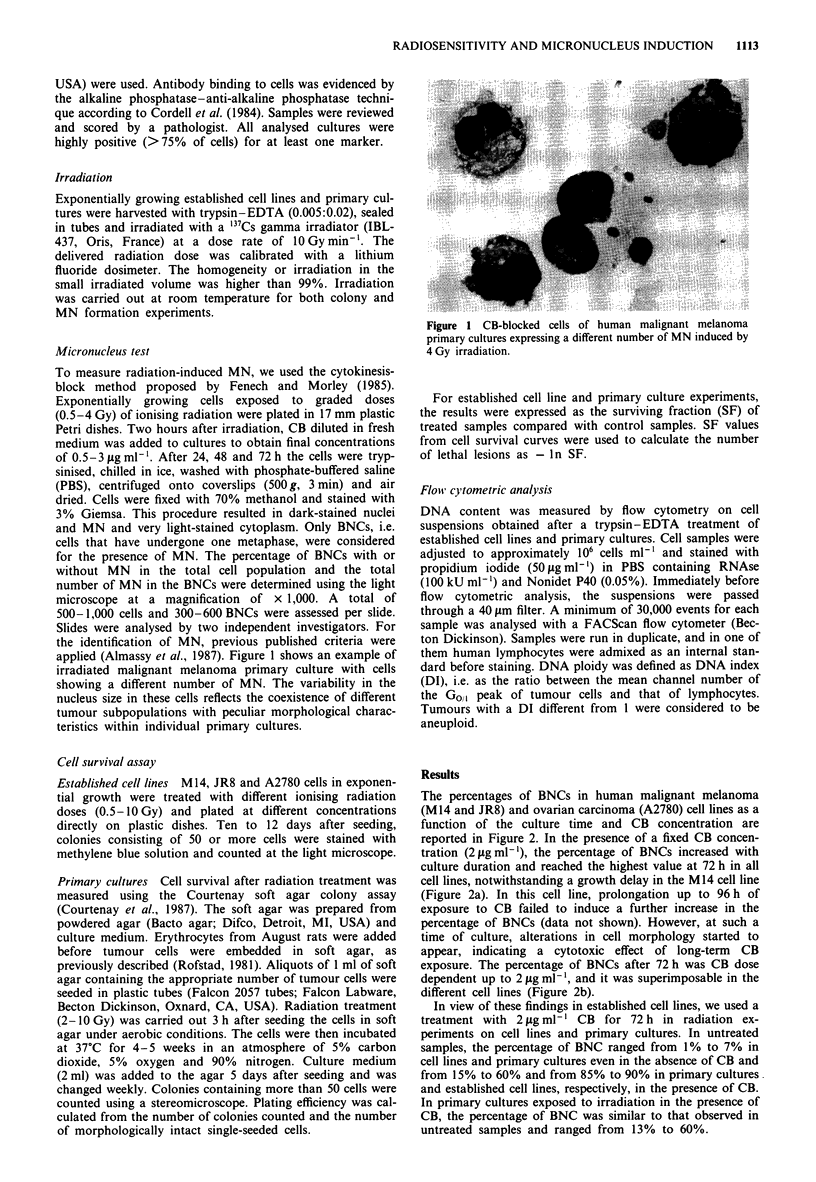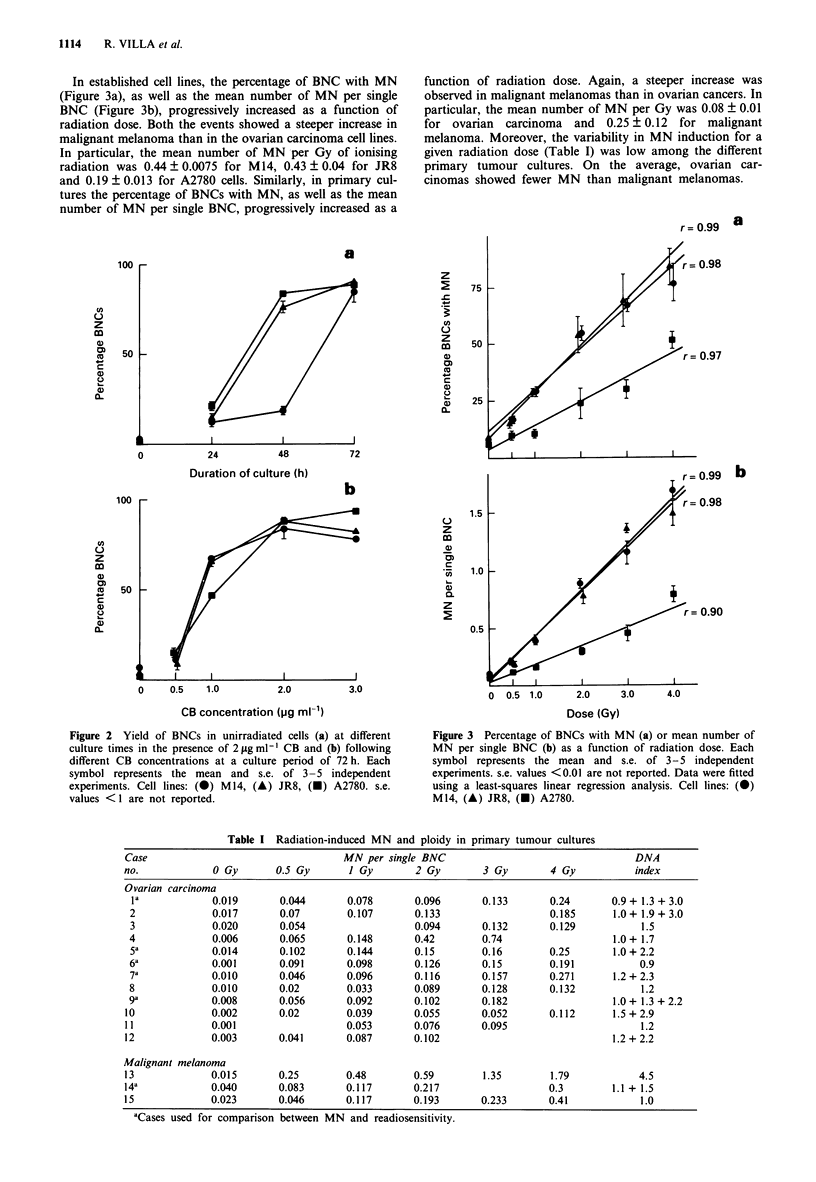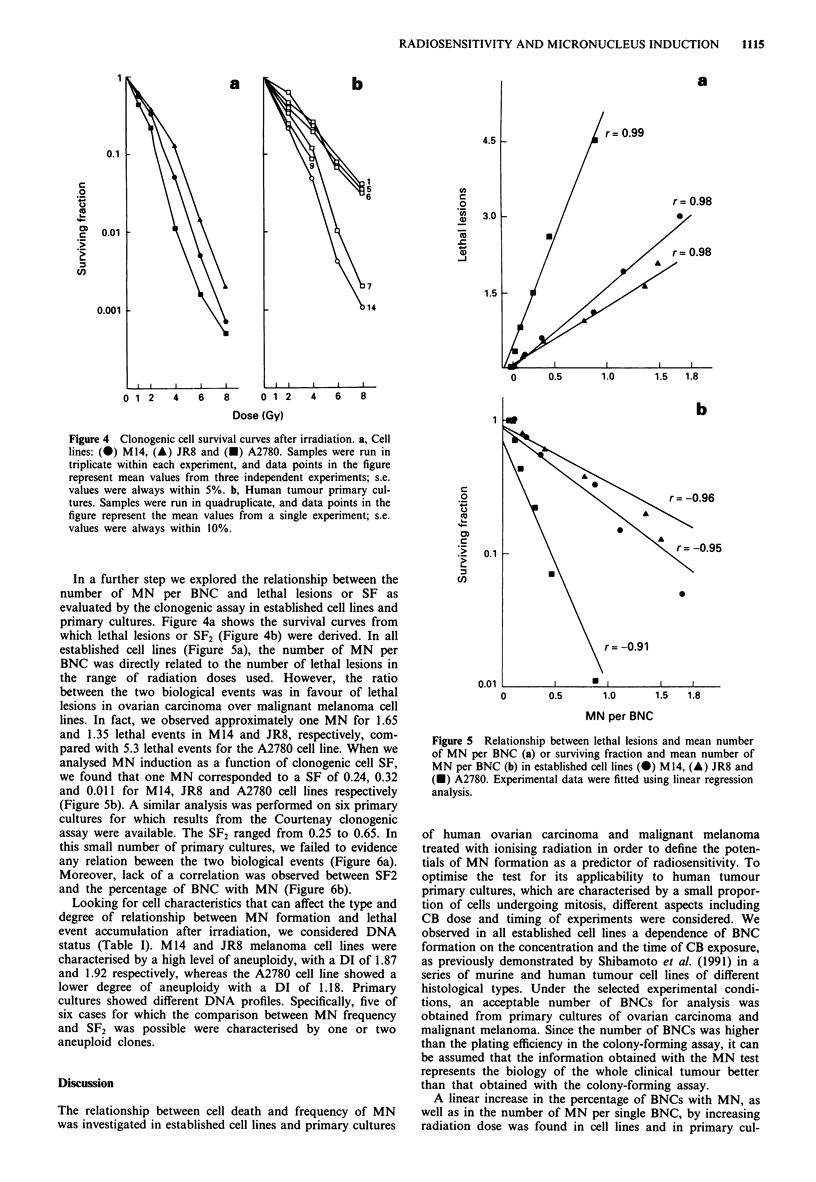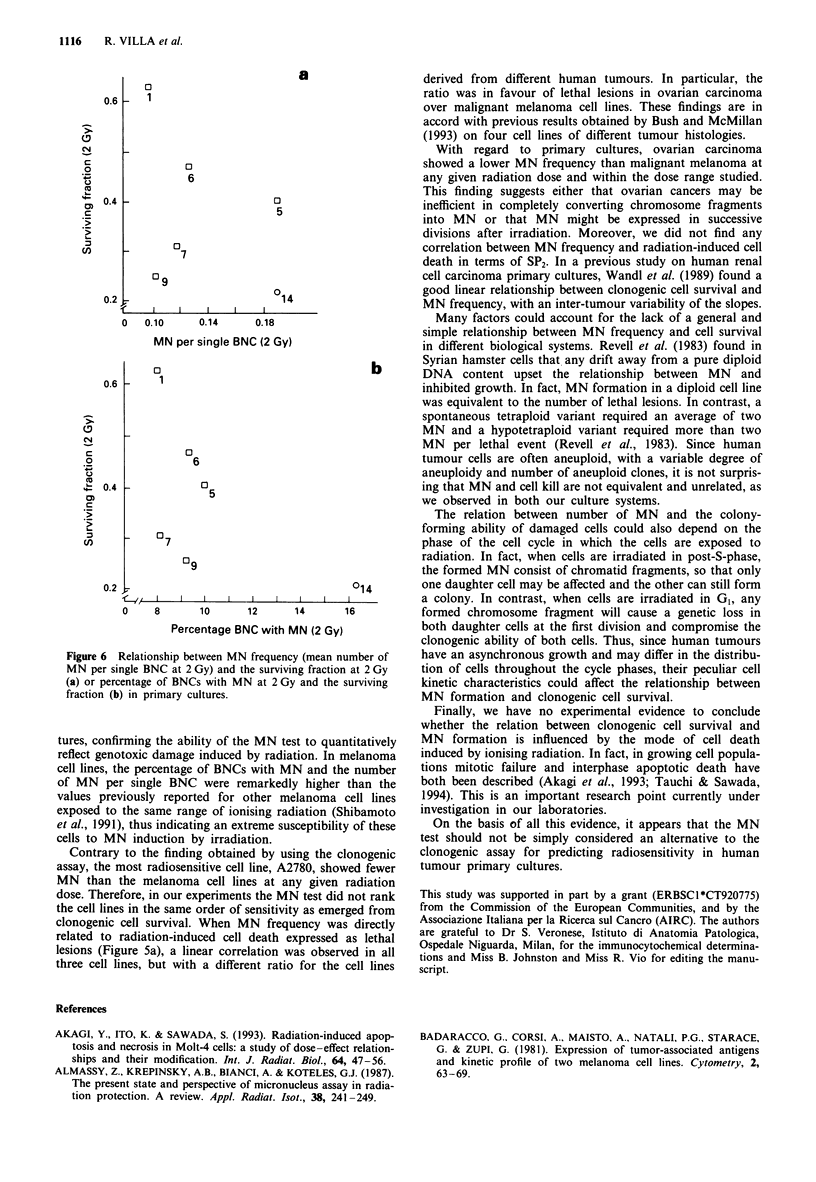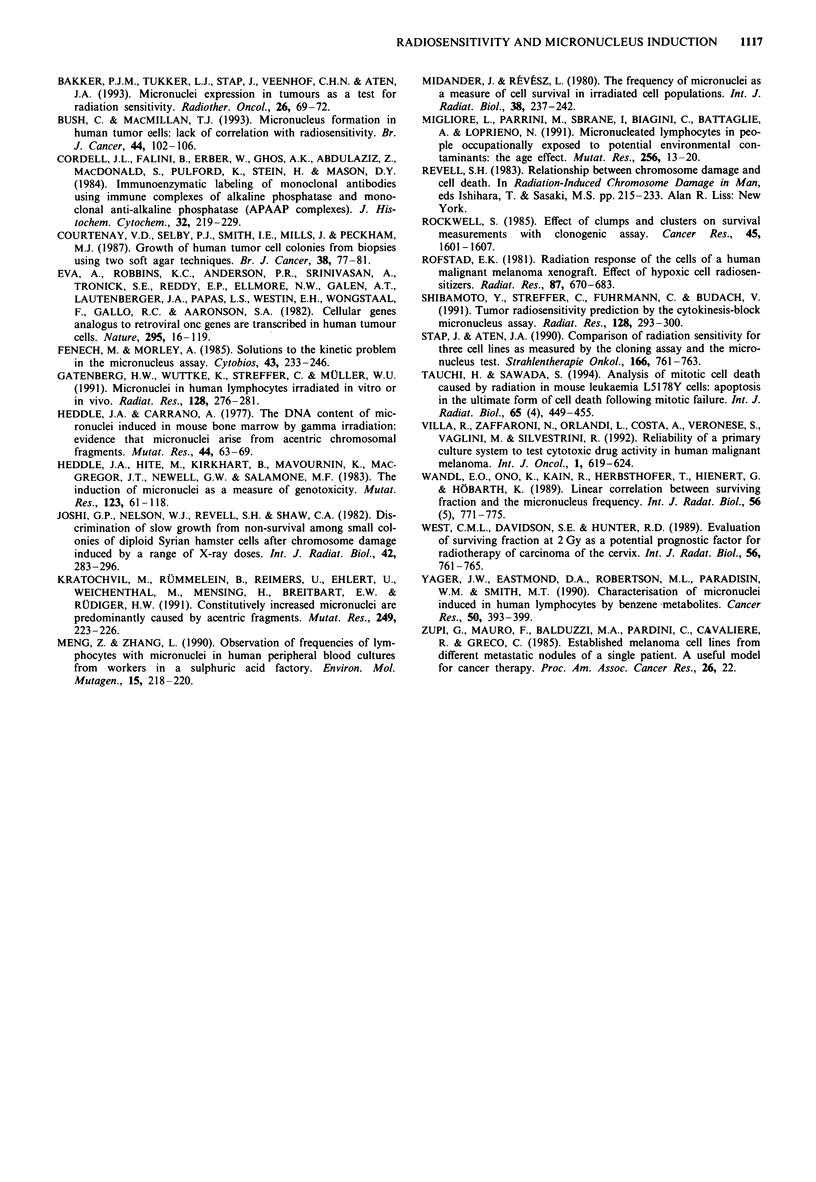# Lack of a correlation between micronucleus formation and radiosensitivity in established and primary cultures of human tumours.

**DOI:** 10.1038/bjc.1994.457

**Published:** 1994-12

**Authors:** R. Villa, N. Zaffaroni, D. Gornati, A. Costa, R. Silvestrini

**Affiliations:** Department of Experimental Oncology C, Istituto Nazionale per lo Studio e la Cura dei Tumori, Milan, Italy.

## Abstract

**Images:**


*Br. J. Cancer* (1994), **70**, 1112–1117 © Macmillan Press Ltd., 1994

### Lack of a correlation between micronucleus formation and radiosensitivity in established and primary cultures of human tumours

R. Villa, N. Zaffaroni, D. Gornati, A. Costa & R. Silvestrini

*Department of Experimental Oncology C, Istituto Nazionale per lo Studio e la Cura dei Tumori, 20133 Milan, Italy.*

**Summary** The radiation-induced genotoxic damage in three established cell lines and 15 primary cultures of human malignant melanoma and ovarian carcinoma showing different radiosensitivity was tested by the cytokinesis-block micronucleus assay. A dose-related increase in micronucleus frequency was observed in all the cell systems. The mean number of micronuclei per Gy of ionising radiation per binucleated cell was respectively $0.44 \pm 0.0075$ and $0.43 \pm 0.04$ for M14 and JR8 malignant melanoma cell lines and $0.19 \pm 0.013$ for the A2780 ovarian cancer cell line. The number of micronuclei did not rank the cell lines in the same order of radiosensitivity as clonogenic cell survival, which showed a surviving fraction at 2 Gy of $0.38 \pm 0.02$ for JR8, $0.34 \pm 0.05$ for M14 and $0.22 \pm 0.007$ for A2780. As regards primary tumour cultures, no correlation was observed between micronucleus induction and surviving fraction at 2 Gy. In conclusion, the discrepancy we observed between micronucleus formation and cell death raises doubts about the potential of the micronucleus assay as a preclinical means to predict radiosensitivity.

The standard assay for radiosensitivity has been for some time the *in vitro* measurement of clonogenic cell survival, and the surviving fraction at 2 Gy ($SF_2$) is still currently investigated as an indicator of clinical tumour radioresponsiveness (West *et al.*, 1989). However, extremely low plating efficiency and clumping artefacts may severely limit the feasibility of the clonogenic assay in human tumours (Rockwell, 1985). It is therefore important to have non-clonogenic assays able to predict radioresponsivity at a preclinical level.

Owing to its simplicity, rapidity and high feasibility, the micronucleus (MN) test has been used for the evaluation of environmental and industrial hazards including ionising radiation (Midander & Revesz, 1980; Heddle *et al.*, 1983; Meng & Zhang, 1990). MN arise from chromatin that fails to be incorporated into nuclei during karyokinesis. Following chromosome damage and spindle dysfunction caused by genotoxic factors (Yager *et al.*, 1990; Gantenberg *et al.*, 1991; Migliore *et al.*, 1991), chromosome fragments or complete chromosomes behave independently of the remaining chromosomes during the division of cells and give rise to MN (Heddle & Carrano, 1977; Kratochvil *et al.*, 1991).

Several investigators have shown a close correlation between genotoxicity defined as MN formation and radiotoxicity (assessed by inhibition on colony formation) in normal and established tumour cell lines (Joshi *et al.*, 1982; Stap & Aten, 1990; Bakker *et al.*, 1993). However, this direct relationship has recently been questioned by Bush and McMillan (1993). Therefore, the potential of the MN test to become a rapid predictive assay should be further investigated. In particular, the feasibility and reliability of the MN test should be directly assessed on primary cultures of human tumours.

In the present study we proposed to investigate the relationship between clonogenic cell kill and MN formation induced by radiation. The study was performed on cell lines and primary cultures of human malignant melanoma and ovarian carcinoma. Since MN arise only at the time of mitosis, and the fraction of mitotic cells may be low or not very high in human tumours, we used the cytokinesis-block MN test (Fenech & Morley, 1985). This assay allows the scoring of MN in binucleated cells (BNCs) that have undergone mitosis and is considered to be much more efficient, rapid and less expertise demanding than the conventional MN test.

Correspondence: R. Silvestrini, Oncologia Sperimentale C, Istituto Nazionale Tumori, Via Venezian 1, 20133 Milan, Italy.
Received 5 April 1994; and in revised form 7 July 1994.

### Materials and methods

### *Drug*

Cytocalasin B (CB) was dissolved in dimethylsulphoxide and stored in aliquots at − 80°C at a concentration of 1 mg ml$^{-1}$. Freshly thawed stock solution was diluted with Hanks' balanced salt solution to produce the required concentration.

### *Cell lines*

Human malignant melanoma (M14, JR8) and ovarian carcinoma (A2780) cell lines were used. Their biological characteristics have been previously described (Badaracco *et al.*, 1981; Eva *et al.*, 1982; Zupi *et al.*, 1985). Cell lines were maintained as a monolayer at 37°C in a 5% carbon dioxide humidified atmosphere in air, using RPMI-1640 medium supplemented with 10% fetal calf serum, 2 μM L-glutamine, 0.25 U ml$^{-1}$ insulin (only for A2780), 100 U ml$^{-1}$ penicillin and 100 μg ml$^{-1}$ streptomycin.

### *Primary cultures*

Tumour specimens were obtained from 15 patients who underwent surgery at the National Cancer Institute of Milan. Samples included three lymph node metastases from malignant melanomas and 12 visceral metastases from ovarian cancers. Immediately following surgery, the samples were placed in cold Hanks' balanced salt solution, trimmed of adipose and necrotic tissue and cut into small fragments. Melanoma fragments were mechanically disaggregated, whereas ovarian cancer fragments were enzymatically digested to obtain a cell suspension, as previously described (Villa *et al.*, 1992). Cell viability, determined by trypan blue dye exclusion, ranged from 15% to 90%, with a median value of 48%.

To obtain primary cultures, $2-5 \times 10^4$ viable cells per cm$^2$ were plated in 7 ml of serum-free Dulbecco's modified Eagle medium and Ham's nutrient mixture F12 (Sigma, St Louis, MO, USA) in plastic flasks and cultured as reported elsewhere (Villa *et al.*, 1992). Cultures were maintained in an incubator (Cytoperm, Hereaus, Hanau, Germany) with 5% carbon dioxide in air at 37°C and 95% relative humidity. To confirm the nature of cells grown in flasks, different monoclonal antibodies with specificity against melanoma (anti-S100, Biogenex Laboratories, San Ramon, CA, USA; and HMB45, Enzo Biochem., New York, NY, USA) and ovarian cancer cells (anti-CA125, Signet Laboratories, Dedham, MA,

USA) were used. Antibody binding to cells was evidenced by the alkaline phosphatase–anti-alkaline phosphatase technique according to Cordell *et al.* (1984). Samples were reviewed and scored by a pathologist. All analysed cultures were highly positive (>75% of cells) for at least one marker.

### *Irradiation*

Exponentially growing established cell lines and primary cultures were harvested with trypsin–EDTA (0.005:0.02), sealed in tubes and irradiated with a $^{137}Cs$ gamma irradiator (IBL-437, Oris, France) at a dose rate of 10 Gy min$^{-1}$. The delivered radiation dose was calibrated with a lithium fluoride dosimeter. The homogeneity or irradiation in the small irradiated volume was higher than 99%. Irradiation was carried out at room temperature for both colony and MN formation experiments.

### *Micronucleus test*

To measure radiation-induced MN, we used the cytokinesis-block method proposed by Fenech and Morley (1985). Exponentially growing cells exposed to graded doses (0.5–4 Gy) of ionising radiation were plated in 17 mm plastic Petri dishes. Two hours after irradiation, CB diluted in fresh medium was added to cultures to obtain final concentrations of 0.5–3 $\mu$g ml$^{-1}$. After 24, 48 and 72 h the cells were trypsinised, chilled in ice, washed with phosphate-buffered saline (PBS), centrifuged onto coverslips (500 *g*, 3 min) and air dried. Cells were fixed with 70% methanol and stained with 3% Giemsa. This procedure resulted in dark-stained nuclei and MN and very light-stained cytoplasm. Only BNCs, i.e. cells that have undergone one metaphase, were considered for the presence of MN. The percentage of BNCs with or without MN in the total cell population and the total number of MN in the BNCs were determined using the light microscope at a magnification of × 1,000. A total of 500–1,000 cells and 300–600 BNCs were assessed per slide. Slides were analysed by two independent investigators. For the identification of MN, previous published criteria were applied (Almassy *et al.*, 1987). Figure 1 shows an example of irradiated malignant melanoma primary culture with cells showing a different number of MN. The variability in the nucleus size in these cells reflects the coexistence of different tumour subpopulations with peculiar morphological characteristics within individual primary cultures.

### *Cell survival assay*

*Established cell lines* M14, JR8 and A2780 cells in exponential growth were treated with different ionising radiation doses (0.5–10 Gy) and plated at different concentrations directly on plastic dishes. Ten to 12 days after seeding, colonies consisting of 50 or more cells were stained with methylene blue solution and counted at the light microscope.

*Primary cultures* Cell survival after radiation treatment was measured using the Courtenay soft agar colony assay (Courtenay *et al.*, 1987). The soft agar was prepared from powdered agar (Bacto agar; Difco, Detroit, MI, USA) and culture medium. Erythrocytes from August rats were added before tumour cells were embedded in soft agar, as previously described (Rofstad, 1981). Aliquots of 1 ml of soft agar containing the appropriate number of tumour cells were seeded in plastic tubes (Falcon 2057 tubes; Falcon Labware, Becton Dickinson, Oxnard, CA, USA). Radiation treatment (2–10 Gy) was carried out 3 h after seeding the cells in soft agar under aerobic conditions. The cells were then incubated at 37°C for 4–5 weeks in an atmosphere of 5% carbon dioxide, 5% oxygen and 90% nitrogen. Culture medium (2 ml) was added to the agar 5 days after seeding and was changed weekly. Colonies containing more than 50 cells were counted using a stereomicroscope. Plating efficiency was calculated from the number of colonies counted and the number of morphologically intact single-seeded cells.

**Figure 1** CB-blocked cells of human malignant melanoma primary cultures expressing a different number of MN induced by 4 Gy irradiation.

For established cell line and primary culture experiments, the results were expressed as the surviving fraction (SF) of treated samples compared with control samples. SF values from cell survival curves were used to calculate the number of lethal lesions as − ln SF.

### *Flow cytometric analysis*

DNA content was measured by flow cytometry on cell suspensions obtained after a trypsin–EDTA treatment of established cell lines and primary cultures. Cell samples were adjusted to approximately $10^6$ cells ml$^{-1}$ and stained with propidium iodide (50 $\mu$g ml$^{-1}$) in PBS containing RNAse (100 kU ml$^{-1}$) and Nonidet P40 (0.05%). Immediately before flow cytometric analysis, the suspensions were passed through a 40 $\mu$m filter. A minimum of 30,000 events for each sample was analysed with a FACScan flow cytometer (Becton Dickinson). Samples were run in duplicate, and in one of them human lymphocytes were admixed as an internal standard before staining. DNA ploidy was defined as DNA index (DI), i.e. as the ratio between the mean channel number of the $G_{0/1}$ peak of tumour cells and that of lymphocytes. Tumours with a DI different from 1 were considered to be aneuploid.

### Results

The percentages of BNCs in human malignant melanoma (M14 and JR8) and ovarian carcinoma (A2780) cell lines as a function of the culture time and CB concentration are reported in Figure 2. In the presence of a fixed CB concentration (2 $\mu$g ml$^{-1}$), the percentage of BNCs increased with culture duration and reached the highest value at 72 h in all cell lines, notwithstanding a growth delay in the M14 cell line (Figure 2a). In this cell line, prolongation up to 96 h of exposure to CB failed to induce a further increase in the percentage of BNCs (data not shown). However, at such a time of culture, alterations in cell morphology started to appear, indicating a cytotoxic effect of long-term CB exposure. The percentage of BNCs after 72 h was CB dose dependent up to 2 $\mu$g ml$^{-1}$, and it was superimposable in the different cell lines (Figure 2b).

In view of these findings in established cell lines, we used a treatment with 2 $\mu$g ml$^{-1}$ CB for 72 h in radiation experiments on cell lines and primary cultures. In untreated samples, the percentage of BNC ranged from 1% to 7% in cell lines and primary cultures even in the absence of CB and from 15% to 60% and from 85% to 90% in primary cultures and established cell lines, respectively, in the presence of CB. In primary cultures exposed to irradiation in the presence of CB, the percentage of BNC was similar to that observed in untreated samples and ranged from 13% to 60%.

In established cell lines, the percentage of BNC with MN (Figure 3a), as well as the mean number of MN per single BNC (Figure 3b), progressively increased as a function of radiation dose. Both the events showed a steeper increase in malignant melanoma than in the ovarian carcinoma cell lines. In particular, the mean number of MN per Gy of ionising radiation was 0.44 ± 0.0075 for M14, 0.43 ± 0.04 for JR8 and 0.19 ± 0.013 for A2780 cells. Similarly, in primary cultures the percentage of BNCs with MN, as well as the mean number of MN per single BNC, progressively increased as a function of radiation dose. Again, a steeper increase was observed in malignant melanomas than in ovarian cancers. In particular, the mean number of MN per Gy was 0.08 ± 0.01 for ovarian carcinoma and 0.25 ± 0.12 for malignant melanoma. Moreover, the variability in MN induction for a given radiation dose (Table I) was low among the different primary tumour cultures. On the average, ovarian carcinomas showed fewer MN than malignant melanomas.

Figure 2 Yield of BNCs in unirradiated cells (a) at different culture times in the presence of 2 μg ml$^{-1}$ CB and (b) following different CB concentrations at a culture period of 72 h. Each symbol represents the mean and s.e. of 3–5 independent experiments. Cell lines: (●) M14, (▲) JR8, (■) A2780. s.e. values < 1 are not reported.

Figure 3 Percentage of BNCs with MN (a) or mean number of MN per single BNC (b) as a function of radiation dose. Each symbol represents the mean and s.e. of 3–5 independent experiments. s.e. values < 0.01 are not reported. Data were fitted using a least-squares linear regression analysis. Cell lines: (●) M14, (▲) JR8, (■) A2780.

**Table I** Radiation-induced MN and ploidy in primary tumour cultures

| *Case no.* | *MN per single BNC* *0 Gy* | *0.5 Gy* | *1 Gy* | *2 Gy* | *3 Gy* | *4 Gy* | *DNA index* |
|---|---|---|---|---|---|---|---|
| *Ovarian carcinoma* | | | | | | | |
| 1[a] | 0.019 | 0.044 | 0.078 | 0.096 | 0.133 | 0.24 | 0.9 + 1.3 + 3.0 |
| 2 | 0.017 | 0.07 | 0.107 | 0.133 | | 0.185 | 1.0 + 1.9 + 3.0 |
| 3 | 0.020 | 0.054 | | 0.094 | 0.132 | 0.129 | 1.5 |
| 4 | 0.006 | 0.065 | 0.148 | 0.42 | 0.74 | | 1.0 + 1.7 |
| 5[a] | 0.014 | 0.102 | 0.144 | 0.15 | 0.16 | 0.25 | 1.0 + 2.2 |
| 6[a] | 0.001 | 0.091 | 0.098 | 0.126 | 0.15 | 0.191 | 0.9 |
| 7[a] | 0.010 | 0.046 | 0.096 | 0.116 | 0.157 | 0.271 | 1.2 + 2.3 |
| 8 | 0.010 | 0.02 | 0.033 | 0.089 | 0.128 | 0.132 | 1.2 |
| 9[a] | 0.008 | 0.056 | 0.092 | 0.102 | 0.182 | | 1.0 + 1.3 + 2.2 |
| 10 | 0.002 | 0.02 | 0.039 | 0.055 | 0.052 | 0.112 | 1.5 + 2.9 |
| 11 | 0.001 | | 0.053 | 0.076 | 0.095 | | 1.2 |
| 12 | 0.003 | 0.041 | 0.087 | 0.102 | | | 1.2 + 2.2 |
| *Malignant melanoma* | | | | | | | |
| 13 | 0.015 | 0.25 | 0.48 | 0.59 | 1.35 | 1.79 | 4.5 |
| 14[a] | 0.040 | 0.083 | 0.117 | 0.217 | | 0.3 | 1.1 + 1.5 |
| 15 | 0.023 | 0.046 | 0.117 | 0.193 | 0.233 | 0.41 | 1.0 |

[a]Cases used for comparison between MN and readiosensitivity.

**Figure 4** Clonogenic cell survival curves after irradiation. **a**, Cell lines: (●) M14, (▲) JR8 and (■) A2780. Samples were run in triplicate within each experiment, and data points in the figure represent mean values from three independent experiments; s.e. values were always within 5%. **b**, Human tumour primary cultures. Samples were run in quadruplicate, and data points in the figure represent the mean values from a single experiment; s.e. values were always within 10%.

**Figure 5** Relationship between lethal lesions and mean number of MN per BNC (**a**) or surviving fraction and mean number of MN per BNC (**b**) in established cell lines (●) M14, (▲) JR8 and (■) A2780. Experimental data were fitted using linear regression analysis.

In a further step we explored the relationship between the number of MN per BNC and lethal lesions or SF as evaluated by the clonogenic assay in established cell lines and primary cultures. Figure 4a shows the survival curves from which lethal lesions or $SF_2$ (Figure 4b) were derived. In all established cell lines (Figure 5a), the number of MN per BNC was directly related to the number of lethal lesions in the range of radiation doses used. However, the ratio between the two biological events was in favour of lethal lesions in ovarian carcinoma over malignant melanoma cell lines. In fact, we observed approximately one MN for 1.65 and 1.35 lethal events in M14 and JR8, respectively, compared with 5.3 lethal events for the A2780 cell line. When we analysed MN induction as a function of clonogenic cell SF, we found that one MN corresponded to a SF of 0.24, 0.32 and 0.011 for M14, JR8 and A2780 cell lines respectively (Figure 5b). A similar analysis was performed on six primary cultures for which results from the Courtenay clonogenic assay were available. The $SF_2$ ranged from 0.25 to 0.65. In this small number of primary cultures, we failed to evidence any relation beween the two biological events (Figure 6a). Moreover, lack of a correlation was observed between SF2 and the percentage of BNC with MN (Figure 6b).

Looking for cell characteristics that can affect the type and degree of relationship between MN formation and lethal event accumulation after irradiation, we considered DNA status (Table I). M14 and JR8 melanoma cell lines were characterised by a high level of aneuploidy, with a DI of 1.87 and 1.92 respectively, whereas the A2780 cell line showed a lower degree of aneuploidy with a DI of 1.18. Primary cultures showed different DNA profiles. Specifically, five of six cases for which the comparison between MN frequency and $SF_2$ was possible were characterised by one or two aneuploid clones.

### Discussion

The relationship between cell death and frequency of MN was investigated in established cell lines and primary cultures of human ovarian carcinoma and malignant melanoma treated with ionising radiation in order to define the potentials of MN formation as a predictor of radiosensitivity. To optimise the test for its applicability to human tumour primary cultures, which are characterised by a small proportion of cells undergoing mitosis, different aspects including CB dose and timing of experiments were considered. We observed in all established cell lines a dependence of BNC formation on the concentration and the time of CB exposure, as previously demonstrated by Shibamoto *et al.* (1991) in a series of murine and human tumour cell lines of different histological types. Under the selected experimental conditions, an acceptable number of BNCs for analysis was obtained from primary cultures of ovarian carcinoma and malignant melanoma. Since the number of BNCs was higher than the plating efficiency in the colony-forming assay, it can be assumed that the information obtained with the MN test represents the biology of the whole clinical tumour better than that obtained with the colony-forming assay.

A linear increase in the percentage of BNCs with MN, as well as in the number of MN per single BNC, by increasing radiation dose was found in cell lines and in primary cul-

**Figure 6** Relationship between MN frequency (mean number of MN per single BNC at 2 Gy) and the surviving fraction at 2 Gy (**a**) or percentage of BNCs with MN at 2 Gy and the surviving fraction (**b**) in primary cultures.

tures, confirming the ability of the MN test to quantitatively reflect genotoxic damage induced by radiation. In melanoma cell lines, the percentage of BNCs with MN and the number of MN per single BNC were remarkedly higher than the values previously reported for other melanoma cell lines exposed to the same range of ionising radiation (Shibamoto *et al.*, 1991), thus indicating an extreme susceptibility of these cells to MN induction by irradiation.

Contrary to the finding obtained by using the clonogenic assay, the most radiosensitive cell line, A2780, showed fewer MN than the melanoma cell lines at any given radiation dose. Therefore, in our experiments the MN test did not rank the cell lines in the same order of sensitivity as emerged from clonogenic cell survival. When MN frequency was directly related to radiation-induced cell death expressed as lethal lesions (Figure 5a), a linear correlation was observed in all three cell lines, but with a different ratio for the cell lines derived from different human tumours. In particular, the ratio was in favour of lethal lesions in ovarian carcinoma over malignant melanoma cell lines. These findings are in accord with previous results obtained by Bush and McMillan (1993) on four cell lines of different tumour histologies.

With regard to primary cultures, ovarian carcinoma showed a lower MN frequency than malignant melanoma at any given radiation dose and within the dose range studied. This finding suggests either that ovarian cancers may be inefficient in completely converting chromosome fragments into MN or that MN might be expressed in successive divisions after irradiation. Moreover, we did not find any correlation between MN frequency and radiation-induced cell death in terms of $SP_2$. In a previous study on human renal cell carcinoma primary cultures, Wandl *et al.* (1989) found a good linear relationship between clonogenic cell survival and MN frequency, with an inter-tumour variability of the slopes.

Many factors could account for the lack of a general and simple relationship between MN frequency and cell survival in different biological systems. Revell *et al.* (1983) found in Syrian hamster cells that any drift away from a pure diploid DNA content upset the relationship between MN and inhibited growth. In fact, MN formation in a diploid cell line was equivalent to the number of lethal lesions. In contrast, a spontaneous tetraploid variant required an average of two MN and a hypotetraploid variant required more than two MN per lethal event (Revell *et al.*, 1983). Since human tumour cells are often aneuploid, with a variable degree of aneuploidy and number of aneuploid clones, it is not surprising that MN and cell kill are not equivalent and unrelated, as we observed in both our culture systems.

The relation between number of MN and the colony-forming ability of damaged cells could also depend on the phase of the cell cycle in which the cells are exposed to radiation. In fact, when cells are irradiated in post-S-phase, the formed MN consist of chromatid fragments, so that only one daughter cell may be affected and the other can still form a colony. In contrast, when cells are irradiated in $G_1$, any formed chromosome fragment will cause a genetic loss in both daughter cells at the first division and compromise the clonogenic ability of both cells. Thus, since human tumours have an asynchronous growth and may differ in the distribution of cells throughout the cycle phases, their peculiar cell kinetic characteristics could affect the relationship between MN formation and clonogenic cell survival.

Finally, we have no experimental evidence to conclude whether the relation between clonogenic cell survival and MN formation is influenced by the mode of cell death induced by ionising radiation. In fact, in growing cell populations mitotic failure and interphase apoptotic death have both been described (Akagi *et al.*, 1993; Tauchi & Sawada, 1994). This is an important research point currently under investigation in our laboratories.

On the basis of all this evidence, it appears that the MN test should not be simply considered an alternative to the clonogenic assay for predicting radiosensitivity in human tumour primary cultures.

This study was supported in part by a grant (ERBSC1*CT920775) from the Commission of the European Communities, and by the Associazione Italiana per la Ricerca sul Cancro (AIRC). The authors are grateful to Dr S. Veronese, Istituto di Anatomia Patologica, Ospedale Niguarda, Milan, for the immunocytochemical determinations and Miss B. Johnston and Miss R. Vio for editing the manuscript.

### References

AKAGI, Y., ITO, K. & SAWADA, S. (1993). Radiation-induced apoptosis and necrosis in Molt-4 cells: a study of dose-effect relationships and their modification. *Int. J. Radiat. Biol.*, **64**, 47-56.

ALMASSY, Z., KREPINSKY, A.B., BIANCI, A. & KOTELES, G.J. (1987). The present state and perspective of micronucleus assay in radiation protection. A review. *Appl. Radiat. Isot.*, **38**, 241-249.

BADARACCO, G., CORSI, A., MAISTO, A., NATALI, P.G., STARACE, G. & ZUPI, G. (1981). Expression of tumor-associated antigens and kinetic profile of two melanoma cell lines. *Cytometry*, **2**, 63-69.

BAKKER, P.J.M., TUKKER, L.J., STAP, J., VEENHOF, C.H.N. & ATEN, J.A. (1993). Micronuclei expression in tumours as a test for radiation sensitivity. *Radiother. Oncol.*, **26**, 69–72.

BUSH, C. & MACMILLAN, T.J. (1993). Micronucleus formation in human tumor cells: lack of correlation with radiosensitivity. *Br. J. Cancer*, **44**, 102–106.

CORDELL, J.L., FALINI, B., ERBER, W., GHOS, A.K., ABDULAZIZ, Z., MACDONALD, S., PULFORD, K., STEIN, H. & MASON, D.Y. (1984). Immunoenzymatic labeling of monoclonal antibodies using immune complexes of alkaline phosphatase and monoclonal anti-alkaline phosphatase (APAAP complexes). *J. Histochem. Cytochem.*, **32**, 219–229.

COURTENAY, V.D., SELBY, P.J., SMITH, I.E., MILLS, J. & PECKHAM, M.J. (1987). Growth of human tumor cell colonies from biopsies using two soft agar techniques. *Br. J. Cancer*, **38**, 77–81.

EVA, A., ROBBINS, K.C., ANDERSON, P.R., SRINIVASAN, A., TRONICK, S.E., REDDY, E.P., ELLMORE, N.W., GALEN, A.T., LAUTENBERGER, J.A., PAPAS, L.S., WESTIN, E.H., WONGSTAAL, F., GALLO, R.C. & AARONSON, S.A. (1982). Cellular genes analogus to retroviral onc genes are transcribed in human tumour cells. *Nature*, **295**, 16–119.

FENECH, M. & MORLEY, A. (1985). Solutions to the kinetic problem in the micronucleus assay. *Cytobios*, **43**, 233–246.

GATENBERG, H.W., WUTTKE, K., STREFFER, C. & MÜLLER, W.U. (1991). Micronuclei in human lymphocytes irradiated in vitro or in vivo. *Radiat. Res.*, **128**, 276–281.

HEDDLE, J.A. & CARRANO, A. (1977). The DNA content of micronuclei induced in mouse bone marrow by gamma irradiation: evidence that micronuclei arise from acentric chromosomal fragments. *Mutat. Res.*, **44**, 63–69.

HEDDLE, J.A., HITE, M., KIRKHART, B., MAVOURNIN, K., MACGREGOR, J.T., NEWELL, G.W. & SALAMONE, M.F. (1983). The induction of micronuclei as a measure of genotoxicity. *Mutat. Res.*, **123**, 61–118.

JOSHI, G.P., NELSON, W.J., REVELL, S.H. & SHAW, C.A. (1982). Discrimination of slow growth from non-survival among small colonies of diploid Syrian hamster cells after chromosome damage induced by a range of X-ray doses. *Int. J. Radiat. Biol.*, **42**, 283–296.

KRATOCHVIL, M., RÜMMELEIN, B., REIMERS, U., EHLERT, U., WEICHENTHAL, M., MENSING, H., BREITBART, E.W. & RÜDIGER, H.W. (1991). Constitutively increased micronuclei are predominantly caused by acentric fragments. *Mutat. Res.*, **249**, 223–226.

MENG, Z. & ZHANG, L. (1990). Observation of frequencies of lymphocytes with micronuclei in human peripheral blood cultures from workers in a sulphuric acid factory. *Environ. Mol. Mutagen.*, **15**, 218–220.

MIDANDER, J. & RÉVÉSZ, L. (1980). The frequency of micronuclei as a measure of cell survival in irradiated cell populations. *Int. J. Radiat. Biol.*, **38**, 237–242.

MIGLIORE, L., PARRINI, M., SBRANE, I, BIAGINI, C., BATTAGLIE, A. & LOPRIENO, N. (1991). Micronucleated lymphocytes in people occupationally exposed to potential environmental contaminants: the age effect. *Mutat. Res.*, **256**, 13–20.

REVELL, S.H. (1983). Relationship between chromosome damage and cell death. In *Radiation-Induced Chromosome Damage in Man*, eds Ishihara, T. & Sasaki, M.S. pp. 215–233. Alan R. Liss: New York.

ROCKWELL, S. (1985). Effect of clumps and clusters on survival measurements with clonogenic assay. *Cancer Res.*, **45**, 1601–1607.

ROFSTAD, E.K. (1981). Radiation response of the cells of a human malignant melanoma xenograft. Effect of hypoxic cell radiosensitizers. *Radiat. Res.*, **87**, 670–683.

SHIBAMOTO, Y., STREFFER, C., FUHRMANN, C. & BUDACH, V. (1991). Tumor radiosensitivity prediction by the cytokinesis-block micronucleus assay. *Radiat. Res.*, **128**, 293–300.

STAP, J. & ATEN, J.A. (1990). Comparison of radiation sensitivity for three cell lines as measured by the cloning assay and the micronucleus test. *Strahlentherapie Onkol.*, **166**, 761–763.

TAUCHI, H. & SAWADA, S. (1994). Analysis of mitotic cell death caused by radiation in mouse leukaemia L5178Y cells: apoptosis in the ultimate form of cell death following mitotic failure. *Int. J. Radiat. Biol.*, **65** (4), 449–455.

VILLA, R., ZAFFARONI, N., ORLANDI, L., COSTA, A., VERONESE, S., VAGLINI, M. & SILVESTRINI, R. (1992). Reliability of a primary culture system to test cytotoxic drug activity in human malignant melanoma. *Int. J. Oncol.*, **1**, 619–624.

WANDL, E.O., ONO, K., KAIN, R., HERBSTHOFER, T., HIENERT, G. & HÖBARTH, K. (1989). Linear correlation between surviving fraction and the micronucleus frequency. *Int. J. Radat. Biol.*, **56** (5), 771–775.

WEST, C.M.L., DAVIDSON, S.E. & HUNTER, R.D. (1989). Evaluation of surviving fraction at 2 Gy as a potential prognostic factor for radiotherapy of carcinoma of the cervix. *Int. J. Radat. Biol.*, **56**, 761–765.

YAGER, J.W., EASTMOND, D.A., ROBERTSON, M.L., PARADISIN, W.M. & SMITH, M.T. (1990). Characterisation of micronuclei induced in human lymphocytes by benzene metabolites. *Cancer Res.*, **50**, 393–399.

ZUPI, G., MAURO, F., BALDUZZI, M.A., PARDINI, C., CAVALIERE, R. & GRECO, C. (1985). Established melanoma cell lines from different metastatic nodules of a single patient. A useful model for cancer therapy. *Proc. Am. Assoc. Cancer Res.*, **26**, 22.